# Ag/MgO Nanoparticles via Gas Aggregation Nanocluster Source for Perovskite Solar Cell Engineering

**DOI:** 10.3390/ma14195507

**Published:** 2021-09-23

**Authors:** Matteo Caleffi, Paolo Mariani, Giovanni Bertoni, Guido Paolicelli, Luca Pasquali, Antonio Agresti, Sara Pescetelli, Aldo Di Carlo, Valentina De Renzi, Sergio D’Addato

**Affiliations:** 1 Dipartimento di Scienze Fisiche, Matematiche e Informatiche, Università di Modena e Reggio Emilia, Via Campi 213/A, 41125 Modena, Italy; vderenzi@unimore.it (V.D.R.); daddato@unimore.it (S.D.); 2 CHOSE—Centre for Hybrid and Organic Solar Energy, Department of Electronics Engineering, University of Rome Tor Vergata, 00133 Rome, Italy; paolo.mariani@uniroma2.it (P.M.); pescetel@uniroma2.it (S.P.); aldo.dicarlo@uniroma2.it (A.D.C.); 3 CNR—Consiglio Nazionale delle Ricerche, Istituto Nanoscienze, Via Campi 213/A, 41125 Modena, Italy; giovanni.bertoni@nano.cnr.it (G.B.); guido.paolicelli@nano.cnr.it (G.P.); 4 IMEM—CNR, Istituto dei Materiali per l’Elettronica ed il Magnetismo, Consiglio Nazionale delle Ricerche, Parco Area delle Scienze 37/A, 43124 Parma, Italy; 5 Dipartimento di Ingegneria E. Ferrari, Università di Modena e Reggio Emilia, Via Vivarelli 10, 41125 Modena, Italy; luca.pasquali@unimore.it; 6 IOM—CNR, Istituto Officina dei Materiali, Consiglio Nazionale delle Ricerche, s.s. 14, Km. 163.5 in AREA Science Park, Basovizza, 34149 Trieste, Italy; 7 Department of Physics, University of Johannesburg, P.O. Box 524, Auckland Park 2006, South Africa; 8 ISM—CNR, Istituto di Struttura della Materia, Consiglio Nazionale delle Ricerche, 00133 Rome, Italy; 9 EN & TECH, Università di Modena e Reggio Emilia, 41125 Modena, Italy

**Keywords:** nanoparticles, Ag, MgO, perovskite solar cells, gas aggregation nanocluster source, localized surface plasmon resonance

## Abstract

Nanocluster aggregation sources based on magnetron-sputtering represent precise and versatile means to deposit a controlled quantity of metal nanoparticles at selected interfaces. In this work, we exploit this methodology to produce Ag/MgO nanoparticles (NPs) and deposit them on a glass/FTO/TiO_2_ substrate, which constitutes the mesoscopic front electrode of a monolithic perovskite-based solar cell (PSC). Herein, the Ag NP growth through magnetron sputtering and gas aggregation, subsequently covered with MgO ultrathin layers, is fully characterized in terms of structural and morphological properties while thermal stability and endurance against air-induced oxidation are demonstrated in accordance with PSC manufacturing processes. Finally, once the NP coverage is optimized, the Ag/MgO engineered PSCs demonstrate an overall increase of 5% in terms of device power conversion efficiencies (up to 17.8%).

## 1. Introduction

Recently, the development of efficient and low-cost photovoltaic technologies has gained increasing attention from the scientific community for satisfying the thirst for clean and sustainably obtained energy. Amongst third generation photovoltaics, perovskite solar cells (PSCs) stand out due to their power conversion efficiency (PCE), which in the past ten years has been boosted from 3.8% in 2009 up to 25.6% in 2021 [1], making them comparable, in terms of PCE, with mature Si solar cells [2]. Such an astonishing result was triggered by the progressive optimization of a PSC structure mainly composed of a perovskite absorber sandwiched between hole and electron selective contacts. Indeed, interfaces between adjacent layers play a crucial role in ruling the device performance and stability, since a charge carrier generated within the perovskite absorber should be efficiently transferred to the selective layers without recombination before being collected at the electrodes [3]. As a matter of fact, the PSC structure should be designed properly with the aid of energy band diagrams to avoid barriers at the interfaces, while adjacent layers should be deposited on top of each other by avoiding pinholes or defects due to the poor adhesion, lattice mismatch, solvent incompatibility and/or layer degradation, which occur in post-deposition processing or when the complete devices are in real working conditions and subject to prolonged illumination, prolonged heating, etc.

In this context, interface engineering (IE) has been proposed as a winning strategy for tuning the interface opto-electronic properties, improving the charge dynamics within the device and eventually enhancing the device performance and stability [4]. IE strategies can be classified into three main groups: (i) looking for alternative selective contact materials on the basis of their morphologies and opto-electronic properties [5]; (ii) modification of the perovskite crystalline structure by finely controlling the crystal growing conditions [6], the perovskite precursor solution with possible doping and by considering a 2D perovskite overlayer atop the 3D perovskite absorber [7]; (iii) surface modification of selective contact (mainly involving TiO_2_ when dealing with the mesoscopic n-i-p structure) [8]. The first strategy needs to completely redesign the device in terms of energy level alignment and to reassess the device lifetime under different stress test conditions. The second strategy requires the optimization of the deposition process for the modified perovskite precursor solution or the fine control of the deposition conditions, as well as an additional production step in the case of a 2D perovskite overlayer. On the contrary, the surface modification of the selective contact where the perovskite layer is deposited could be a viable route for improving the PSC performance without modifying the perovskite precursor composition and eventually its deposition conditions. Moreover, any treatment needed to achieve the selective contact surface modifications (i.e., thermal annealing, UV–ozone, etc.) is only limited by the material constituting the electrode. For example, in the case of mesoscopic PSCs, the usually employed TiO_2_ layer can be subjected to severe treatment such as high temperature (i.e., in the case of lithium salt treatment) [9], aggressive chemical baths (i.e., TiCl_4_ treatments) [10] or harsh conditions typically achieved using invasive implantation or deposition techniques (i.e., ion implantation or atomic layer deposition (ALD)) [11]. The mesoscopic structure is thus the best candidate to select for testing IE based on surface modification, even considering that the highest efficiencies ever certified for PSCs were demonstrated by employing a mesoscopic structure [1]. Considering a mesoporous TiO_2_ (m-TiO_2_) layer, IE has been clearly demonstrated as an effective strategy for improving the PSC performance and stability when employing several 2D materials [12] such as graphene [13,14,15] and its derivatives [16,17,18] as well as transition metal carbides or nitrides commonly known as MXenes [19,20]. Notably, the proper 2D materials can be chosen from a wide group (more than 2000 bi-dimensional materials are available), with exceptional opto-electronic properties due to the quantum confinement in the third dimension, that could even be tuned by proper chemical modification or edge modification. Moreover, 2D materials can be produced and dispersed in several solvents by employing a liquid-phase exfoliation (LPE) technique [21] and the production process can be scaled up by employing the recently developed wet-jet milling techniques [22]. However, due to the simplicity of solution-based production processes, 2D materials are usually in the form of a few layers while the monolayer counterpart, desirable for the ideal charge transport properties, is only achieved by employing costly and time-consuming techniques such as chemical vapor deposition (CVD). The impossibility to finely control the number of the constituting layers for 2D material flakes still represents the main drawback in terms of reproducibility and production process yield. Alternatively to 2D materials, the modification of PSC layers can be achieved by employing nanoparticles (NPs), properly designed in terms of chemical composition and size [23]. Indeed, coupling of NPs with photoactive layers is currently an extremely promising route to enhance device performances in photocatalysis [24] and solar energy conversion applications [25,26,27,28,29,30,31]. NP incorporation in photovoltaic (PV) devices has indeed proved successful in increasing their efficiency in a number of cases [27,28,29,30,31], their effectiveness being related to different mechanisms, such as plasmon-enhanced absorption, increased optical path length inside the active layer due to the light scattering effect [32,33], hot-electron injections [34,35,36] and hot spots [37].

In order to be exploited for PV applications, it is important to separate metal NPs from the active layer with an insulating/semiconducting layer, chosen to guarantee: (i) effective hindering of charge recombination at the metal surface; (ii) chemical protection of the metal core, preventing NP aggregation [38] as well as oxidation processes occurring upon device fabrication and functioning, especially when the NPs are exposed to air or other species in the surrounding environment [39]; (iii) thermal stability. On the other hand, the protective layer/shell optical properties and thickness should be chosen to minimize its light absorption and to guarantee the closest possible proximity between the NPs and the active layer.

Furthermore, core–shell NPs allow more flexibility in engineering the optical and electronic properties of the resulting nanostructures, by changing both the shell material and the core–shell size ratio. Recently, improved PSC performance has also been achieved by metal@core–shell NP incorporation at the interface between the photoactive layer (i.e., hybrid organic inorganic lead halide perovskite) and the electron transport layer (ETL) [40,41,42], showing a remarkable relative increase in the PCE values, from 5% up to 45%, with respect to the pristine devices.

These promising results were obtained using NPs with different materials [31,42,43,44] and different geometries [37,45,46]. Among possible metals, silver (Ag) is considered the most promising material for photoactive applications, as it displays a strong absorption in the UV–visible range [47] and a lower manufacturing cost compared with its nearest competitors, gold (Au) and platinum (Pt). As far as shell materials are concerned, most of the studies related to Ag NPs report the use of TiO_2_ or SiO_2_ shells, and chemical wet methods for both NP@core–shell synthesis and the subsequent deposition [48]. Though chemical synthesis guarantees high production outputs and scalability, in some cases, there may be limits to the choice of protective materials and their thickness. For instance, MgO is a very promising alternative shell material due to its wide energy gap (E_g_ = 6 eV) [49], low absorption coefficient in the UV–Vis region, outstanding chemical inertness, high temperature stability and efficacy in shielding metal NPs from the surrounding environment [50]. Despite these characteristics, proper Ag@MgO NPs are notably difficult to obtain by chemical synthesis in liquid solution, due to the incompatibility between Ag NPs and MgO synthesis techniques and the high tendency of MgO to aggregate and form thermodynamically stable films. These difficulties have to date hindered the use of Ag@MgO NPs in photovoltaic applications. A recent study [51] highlights the possibility for a combined use of Au NPs and a MgO passivation film, in particular by incorporating Au NPs into an m-TiO_2_ layer and depositing a MgO film on the Au NP-modified mesoporous TiO_2_ via wet spinning and pyrolysis of magnesium salt. The study demonstrates that the combination of metal NPs and magnesia is an effective and functional blend that can be exploited to design hybrid organic–inorganic perovskite photovoltaic devices with enhanced characteristics, reaching a PCE improvement of about 34% with respect to the pristine device, therefore validating the potential of MgO as outstanding material for PV applications. In addition, MgO has been used as a hole-blocking layer between TiO_2_ ETL and the perovskite, improving the overall performances of solar devices by about 30% in terms of PCE enhancement [52].

In this work, we exploit the extremely versatile and clean physical synthesis methodology to introduce Ag/MgO nanoparticles in a mesoscopic perovskite-based solar device, investigating their in situ properties and their overall effect on the device performance. In more detail, Ag NPs are grown through magnetron sputtering and gas aggregation, deposited in a high vacuum chamber on a glass/FTO/c-TiO_2_/m-TiO_2_ substrate. The protective MgO ultrathin film is introduced with sequential layer deposition [53,54] or co-deposition methods [55,56], by thermally evaporating Mg in an oxygen environment [50], inside an ultra-high vacuum (UHV) chamber. The system morphology and structure are then thoroughly characterized by means of X-ray photoemission spectroscopy (XPS), scanning electron microscopy (SEM), atomic force microscopy (AFM) and transmission electron microscopy (TEM), while its UV–Vis optical properties are investigated by means of polarized reflectivity and transmissivity measurements. Subsequent deposition of the perovskite active layer and fabrication of the final devices allow us to test the cell electrical performance, showing an improvement in current densities in the case of 1.5% Ag/MgO NP loading. As the main result, this study proves the feasibility of physical deposition methods of plasmonic NPs for possible application in PSCs. It therefore paves the way to explore different combinations of metal cores and protective shell or layer materials, aiming for the fabrication of solar devices with increased current density and efficiency.

## 2. Materials and Methods

Mesoporous titania paste (30 NRD), formamidinium iodide (FAI) and methylammonium bromide (MABr) were purchased from GreatCell SolarDyesol^®^. Lead(II) iodide (PbI_2_), and lead(II) bromide (PbBr_2_) were purchased from TCI. Cesium iodide (CsI) was purchased from GmbH. 2,20,7,70-tetrakis-(N,N-dip-methoxyphenylamine)9,9′-spirobifluorene (spiro-OMeTAD) was purchased from Borun, and cobalt(III) FK209 was purchased from Lumtec.

Titanium(IV) isopropoxide (TTIP), diisopropoxytitanium bis(acetylacetonate) (Ti(AcAc)_2_), acetyl acetone (AcAc), acetylacetone, ethanol (EtOH), 2-propanol (IPA), acetone, dimethylformamide (DMF), dimethyl sulfoxide (DMSO), acetonitrile (ACN), tert-butylpyridine (tBP), chlorobenzene (CB) and lithium bis(trifluoromethanesulfonyl)imide (Li-TFSI) were purchased from Sigma-Aldrich.

Patterned fluorinated doped tin oxide (FTO)-coated glasses (Pilkington, 7 Ωcm^−1^) were washed with deionized water and soap, then cleaned first with a cycle of ultrasonic bath in acetone, then with ethanol, for 10 min at each stage. The compact TiO_2_ (c-TiO_2_) layer (around 50 nm) was deposited by a spray pyrolysis technique at 460 °C. The sprayed solution consists of 0.16 M Ti(AcAc)_2_ and 0.4 M AcAc in EtOH. Subsequently, the TiO_2_ mesoporous (m-TiO_2_) solution was prepared using m-TiO_2_ paste (30 NRD) in EtOH (1:5 wt/wt). The resulting m-TiO_2_ solution was deposited on the substrate by spin coating at 3000 rpm for 20 s with subsequent annealing in air for 30 min at 480 °C, resulting in a mesoporous layer with thickness of 120 nm and roughness around 30–40 nm. The engineered mesoscopic substrates were then transferred in an inert environment (nitrogen-filled glovebox) to complete the device realization.

The deposition of Cs_0.05_(MA_0.17_FA_0.83_)_95_Pb(I_0.83_Br_0.17_)_3_ was performed in nitrogen atmosphere following the antisolvent procedure, as detailed in our previous work [57,58,59]. A solution of PbI_2_, PbBr_2_, MABr, FAI and CsI in a solvent mixture of DMF and DMSO with a 4:1 ratio (v:v), respectively, was deposited by spin coating on top of the aforementioned substrates, following a sequential program at 1000 and 5000 rpm for 10 and 30 s, respectively. During the second spin stage, 150 μL of chlorobenzene was quickly dropped on the rotating substrate at 7 s to the end of the program. Then, the substrates were immediately annealed at 100 °C for 1 h. Afterwards, spiro-OMeTAD (73.5 g·L^−1^ in CB solution doped with TBP (26.7 µL·mL^−1^)), LiTFSI (16.6 µL·mL^−1^) and a Co complex (7.2 µL·mL^−1^) were spin coated at 2000 rpm for 20 s. The devices were then finalized by thermally evaporating a gold counter electrode, with a thickness of around 100 nm, in high vacuum conditions (around 10^−6^ mbar), with an active area of 0.1 cm^2^, determined by a black mask applied on the device backside.

The flat TiO_2_ substrate (made of SiO_2_/TiO_2_) was produced at a high deposition rate (tens of nm per minute) by reactive DC-magnetron sputtering from metal targets (Ti) on a SiO_2_ substrate. The overall synthesis and deposition of Ag/MgO NPs was executed inside a UHV chamber connected to a nanocluster source (a complete description of the system can be found elsewhere in previous works [60]). The Ag NP deposition was performed using a nanocluster source, composed of a magnetron (NC200U, Oxford Applied Research), connected with a quadrupole mass filter (QMF). The deposition of MgO was realized by thermally evaporating Mg in an O_2_ atmosphere with similar procedures used elsewhere [50,61]. The deposition rate of Ag and Mg was carefully monitored and tuned using a quartz microbalance. For the experiments reported in this work, Ag NPs were produced with a magnetron discharge power P = 50 W and Ar flow value set at f = 60 sccm. The O_2_ partial pressure was adjusted in order to obtain the right proportion of Mg to O_2_ to form MgO. Typical O_2_ partial pressure was P_O2_ = 3 × 10^−7^ mbar. The Ag NP deposition rate, expressed in thickness of a continuum film with bulk Ag density per unit time, varied between 1 and 0.3 Å/min. MgO deposition rate varied between 10 and 12 Å/min. The amount of deposited Ag NPs is given in this work in terms of surface coverage and for MgO in terms of nominal thickness of an equivalent continuous film with the same density as bulk rock salt MgO. The size distribution of the deposited nanoparticles was estimated ex situ with SEM and TEM. The prepared samples were characterized by SEM using a Nova Nano SEM450 (FEI Company-Bruker Corporation,5350 NE Dawson Creek Drive Hillsboro, Hillsborough, OR, USA).

The SEM column is equipped with a Schottky field-emission gun (SFEG) and it can achieve a resolution of 1.4 nm in low-voltage (1 kV) operation. The TEM measurements were carried out with a TALOS F200S G2 (Thermo-Fisher Scientific,168 Third Avenue Waltham, MA, USA) equipped with a Schottky Field Emitter (80–200 keV) operating in TEM and STEM mode, and a double silicon drift detector (SDD) for energy dispersive X-ray spectroscopy (EDXS). For a better visualization and quantification of the EDXS chemical maps (especially for the low Mg signal), we performed a denoising of the spectra based on principal component analysis (PCA) [62]. The average height distribution of NP films was estimated with AFM (NTEGRA AURA model, NT-MDT).

Thermal annealing for the evaluation of the stability of NPs was performed ex situ and verified with XPS using Al Kα photons and a hemispherical electron analyzer in normal emission geometry. The optical measurements were performed ex situ using a linearly polarized *s* radiation, with an angle of 45° between sample and incident beam. The UV–Vis experiment system architecture was composed of an Ocean Optics DH-20000-BAL light source, the emitted radiation wavelength was between 200 and 1050 nm; the polarizers and the HR4000CG-UV-NIR grating monochromator were purchased from Ocean Optics and furnished with CCD detectors. The J-V characterization of the PSCs was performed with a customized PXI (National Instruments)-based platform (Arkeo). The instrumental setup was composed of a 4-channel source-meter (NI PXIe 4141) and a power supply (NI PXIe 4112) used to deliver high-power white LED (Bridgelux-50C10K0, 5000 Kelvin). The 1 SUN (100 mW·cm^−2^) equivalent incident power was then calibrated with a certified reference Si cell (RERA Solutions RR-1002) through the Mismatch Factor [63,64]. IPCE spectra acquisition were realized using a homemade setup with a monochromator (Newport, mod. 74000, Newport Corporation 1791 Deere Avenue Irvine, CA, USA) coupled with a xenon lamp (Oriel Apex, Newport Corporation 1791 Deere Avenue Irvine, CA, USA) and a source meter (Keithley, mod. 2420), controlled by a home-made LabVIEW program for acquiring spectra [65].

**Figure 1:**
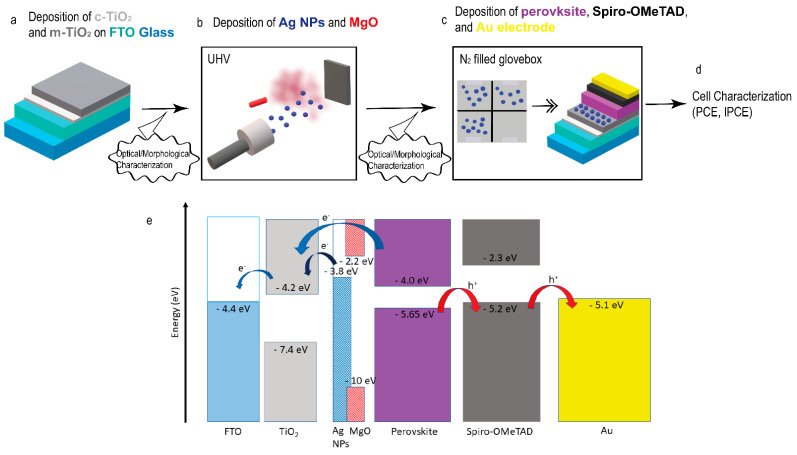
(**a**) Representation of the initial substrate, from bottom to the top: conductive glass substrate (blue) (2.2 mm) covered with an FTO layer (aquamarine) (around 700 nm), compact TiO_2_ layer (light gray) (around 50 nm), mesoporous TiO_2_ layer (gray) (around 150 nm). (**b**) Scheme of the NP (dark blue) synthesis and deposition with MgO (red) on top of the previously realized m-TiO_2_ layer. (**c**) Top view scheme of the experimental device until the m-TiO_2_ layer (see the left part of scheme c), containing four independent cells, three of them with NPs and one used as the reference cell, while the right part of scheme c represents the addition of the perovskite layer (violet) (around 400 nm), Spiro-OMeTAD layer (black) (250 nm) and gold layer (yellow) (around 100 nm); NPs are localized at the interface between the perovskite and mesoporous TiO_2_ layer. The dimensions of each layer displayed here are not to scale. (**d**) Final step of characterization of the engineered solar devices. (**e**) Energy band diagram of the device. The work function values for FTO, TiO_2_ have been taken from references [66,67]. The highest occupied molecular orbital (HOMO) and lowest unoccupied molecular orbital (LUMO) levels for perovskite have been taken from reference [68]. The energy band edge positions for Spiro-OMeTAD have been taken from reference [69]. The energy band values for Ag NPs and MgO are taken from reference [70] and [71], respectively. The depicted energy values are relative to the vacuum level. Blue and red arrows show the direction of electron and hole motion, respectively.

Figure 1 displays a schematic representation of the experimental steps adopted for the realization of every layer of the PSCs and the physical deposition of Ag/MgO NPs, along with the corresponding energy band diagram.

## 3. Results and Discussion

**Figure 2:**
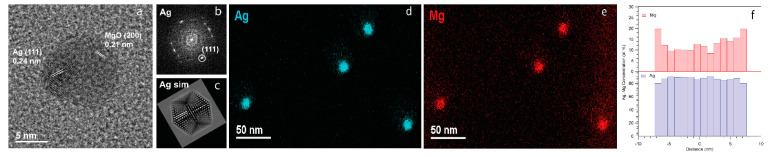
(**a**) Bright-field HRTEM image of an Ag/MgO NP. The lattice fringes from Ag (111) planes are shown in the left part of the NP. Planes assigned to MgO (200) are shown in the top right of the NP image. (**b**) FFT image from the Ag domain, and (**c**) simulated image from an Ag ideal icosahedral domain oriented along the 2-fold axis. (**d**) EDXS map of Ag-L edge intensity (cyan), (**e**) EDXS map of Mg-K edge intensity (red). (**f**) EDXS profile for Ag and Mg content (atomic %) from a single NP.

The formation of Ag/MgO NPs was firstly assessed by TEM, depositing 5 nm (nominal thickness) Ag nanoclusters and 0.8 nm MgO via co-deposition [61] on a TEM grid. The main information of the NP structure was obtained from a high-resolution, bright-field TEM image of a single NP, as shown in Figure 2a. Lattice fringes are visible across almost all the particle, although in some areas they are confused, probably because of the contributions from both the Ag core and MgO protective layer in an almost spherical NP. In particular, in the region delimited by the white square, the fringes can be assigned to an icosahedral-type structure in 2-fold orientation, as can be seen from the simulation in Figure 2c. Ag (111) planes are quite evident (see the fast Fourier transform (FFT) image in Figure 2b), and the analysis gives an interplanar distance of 0.238 nm. The occurrence of the icosahedral shape in physically synthesized face-centered cubic (fcc) metals clusters and NPs—such as bare Ag, Ni and FePt—has been previously reported [50,60,61]. The physical reason for this arrangement has been ascribed to the kinematics of the cluster formation [72,73,74,75] in the aggregation region of the NP source, in particular to the cooling velocity during the collision process with the inert gas atoms. The cluster freezing favors formation of multi-twinned domains in the nascent nanocrystal, which gives rise to metastable structures deviating from the Born-Wulff construction [72,73,74,75]. It can therefore be concluded that the structure of the Ag core, consisting of a multi-twinned McKay icosahedron [73], is preserved during the formation of the MgO film. In the top-right region of the NP, image reflections are visible with a corresponding interplanar distance d = 0.213 nm, which can be assigned to (200) MgO planes. This confirms that MgO covers, at least partially, the Ag core.

In Figure 2d, the EDXS map corresponding to the Ag-L (cyan) edge clearly reveals a few rounded Ag NPs, whose diameter is estimated to be around (10.0 ± 0.6) nm. The Mg map (Figure 2e) provides evidence of the preferential adsorption of MgO on top of Ag NPs, while the EDXS line profile from a single NP reported in Figure 2f shows that Mg has a higher concentration at the edge of the NP, as previously found in similar systems [61] and as expected by a thin capping layer.

It has been demonstrated that the morphological properties of physically synthesized NPs may be significantly influenced by the substrate on which they are deposited [76,77]. For this reason, it is important to fully characterize the Ag/MgO directly on the technologically relevant substrate, i.e., the glass/FTO/c-TiO_2_/m-TiO_2_ (where c-TiO_2_ is a compact titanium dioxide layer, while m-TiO_2_ is the mesoporous top layer) used in fabrication of PSCs [78]. In this case, the MgO capping layer was sequentially deposited on top of NPs, following the procedures given in reference [50]. The effectiveness of this approach relies on the low sticking coefficient of MgO on TiO_2_, as compared to that on Ag. This has been preliminarily proved through XPS; after a nominal deposition of 6 Å MgO—as evaluated by a calibrated quartz microbalance—the amount of Mg deposited on a bare TiO_2_ substrate without NPs—as estimated from XPS quantitative analysis (see Figure S1 in Supplementary Materials)—was 0.1 Å, indicating a MgO sticking coefficient on TiO_2_ substrate of less than 2%. This finding safely allows us to consider the MgO capping layer to cover only the deposited Ag NPs, for relatively low NP coverages, while leaving the bare TiO_2_ substrate essentially unaffected.

**Figure 3:**
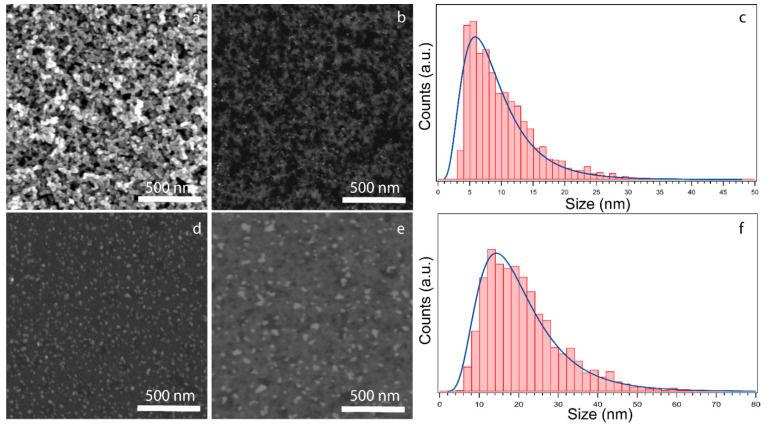
(**a**) SE SEM image of a typical mesoporous TiO_2_ sample, (**b**) BSE SEM image of 1.5% coverage in Ag/MgO sample on m-TiO_2_, (**c**) size distribution resulting from analysis of Figure 3b corresponding to 1.5% NP coverage, (**d**) SE SEM image of Ag/MgO NPs with 10% coverage on a flat TiO_2_ substrate, (**e**) BSE SEM image of 10% coverage of Ag/MgO NPs on m-TiO_2_, (**f**) size distribution resulting from analysis of Figure 3d.

The morphology of Ag/MgO NPs deposited on TiO_2_ substrates was investigated by SEM. As shown in Figure 3a, the substrate top layer is an m-TiO_2_ film characterized by a mesoporous morphology, with pore dimensions of a few tens of nanometers, i.e., comparable with the NP diameter. For this reason, we acquire SEM images using the backscattered electron signal (BSE), which, as shown in Figure 3b,e for 1.5% and 10% coverage, respectively, allows us to single out the heavier Ag cluster (Z = 49) from lighter TiO_2_ substrate and to statistically analyze their size distribution [79], as shown in Figure 3c. The obtained NP size distribution is fitted with the log-normal function described by O’Grady et al. [80], finding an average NP diameter d = 8 nm, with an FWHM of 7 nm for the 1.5% coverage case. For higher coverages, the complicated morphology of the mesoporous films may introduce an overestimation of the NP dimensions, as NPs which are actually on different terraces may improperly appear as single larger aggregates. For this reason, a more thoroughly analysis for 10% coverage was performed using a flat, but chemically equivalent, TiO_2_ substrate, grown on SiO_2_, as shown in Figure 3d. The resulting size distribution gives d = 13 nm and FWHM increases to 17 nm, for the 10% coverage (Figure 3f).

**Figure 4:**
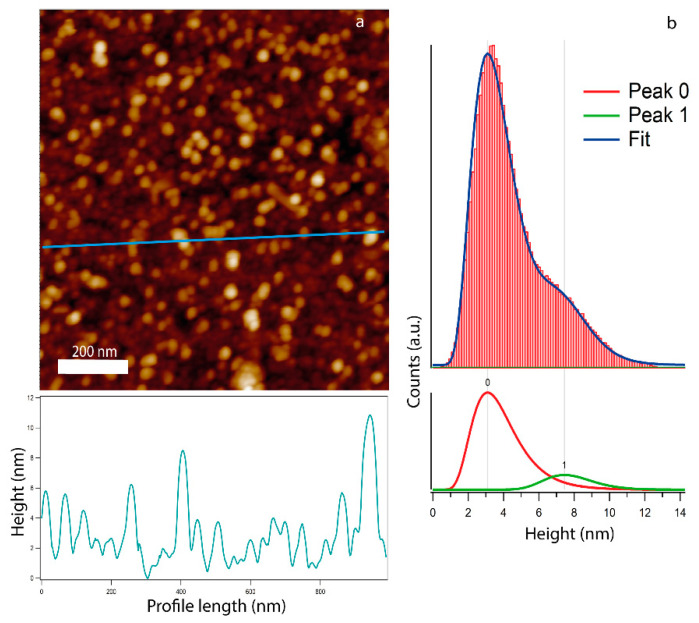
(**a**) AFM image of Ag/MgO NPs on flat TiO_2_ substrate with a profile line at the bottom, (**b**) AFM analysis of the resulting height histogram distribution, red curve (peak 0) is the fitting component corresponding to the substrate roughness, while the green curve (peak 1) is the fitting component corresponding to the height distribution of the Ag/MgO NPs.

Deposition on the flat TiO_2_ substrate also allows us to investigate the NP height distribution by means of AFM topography. In Figure 4, the topography of the Ag/MgO on TiO_2_ is reported (Figure 4a), along with the relative height distribution (Figure 4b). The latter appears clearly as bimodal and it is fitted with two log-normal components: the first component (peak 0) can be ascribed to the surface roughness of TiO_2_ film bare areas with an average height value <h_0_> = 3 and FWHM = 2 nm (as shown in Figure S2, the bare TiO_2_ substrate is characterized by a surface roughness of 3 nm), while the second component (peak 1) corresponds to the NP height distribution, with mean value <h_1_> = 7.3 and FWHM = 2 nm (Figure 4b). A fair estimate of the average NP height can be given by subtracting the average values of the two components, resulting in a value <h> = 4 nm (see also references [81,82]).

Assuming that the NP height does not strongly depend on coverage, for relatively low coverages, and combining SEM and AFM information on size (d) and height (h) distribution, respectively, we can estimate the Ag/MgO NP aspect ratio with the formula AR = d/h. The mean AR value varies from ~2 to 3, upon increasing coverage from 1.5% to 10%; the origin of this flat shape may be associated with both shape deformation as a result of the interaction of the NPs with the surface [81,82] and the formation of small aggregates [50].

**Figure 5:**
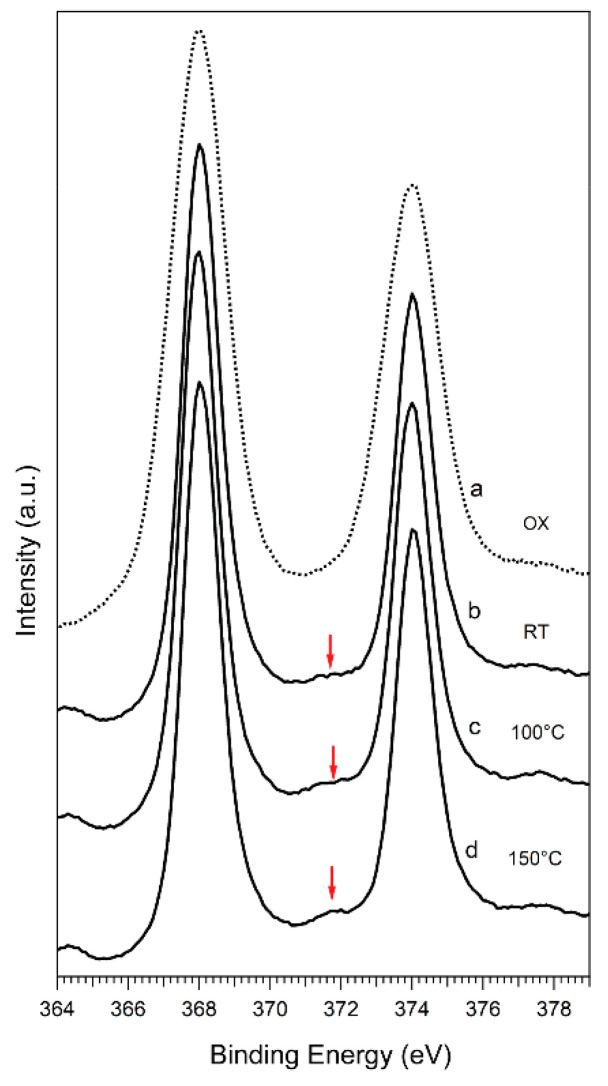
XPS (**a**) Ag 3d core level of bare Ag NPs after air exposure, (**b**) Ag 3d core levels at room temperature (RT), (**c**) Ag 3d core levels at 100 °C and (**d**) Ag 3d core level at 150 °C. The system used was glass/FTO/c-TiO_2_/m-TiO_2_/Ag NPs/MgO. The red arrows highlight the plasmonic features.

The thermal stability of the Ag/MgO-engineered substrates is an extremely relevant aspect to be considered when photovoltaic applications are pursued. Indeed, a temperature as high as 100 °C is reached during the formation of the perovskite layer on top of the functionalized substrate; moreover, the environmental heat generated during PSC working conditions may also increase the temperature well above room temperature [83]. We have therefore tested the thermal stability of the functionalized substrate by means of XPS, upon annealing up to T = 150 °C in UHV (base pressure 5 × 10^−10^ mbar). For this experiment, a glass/FTO/c-TiO_2_/m-TiO_2_ substrate was functionalized with 15% coverage of Ag NPs and covered with 1 nm (nominal thickness) of MgO. It was exposed to air for 10 min during the transfer to another UHV system, equipped with a sample heater and an XPS instrument. Figure 5 shows the Ag 3d core level spectra acquired at room temperature and after annealing at T = 100 °C and T = 150 °C for 20 min, respectively. A spectrum taken on a bare Ag NP sample after exposure to air for 30 min (OX) at room temperature is also shown for comparison. In the case of bare Ag NPs, air exposure completely quenches the plasmonic loss at 372 eV, while the Ag 3d peaks become broader. In the case of Ag/MgO NPs instead, the plasmonic loss is clearly visible, while no oxidized component appears, indicating that MgO is positively acting as a proper capping material in protecting the metal core from oxidation. Furthermore, upon annealing, the Ag plasmonic feature is preserved, proving that the functionalized substrates are stable up to 150 °C, with Ag in its metallic phase. In addition, the Ag and Mg core level intensities remain essentially constant during the annealing, showing that the system is stable in this temperature range.

**Figure 6:**
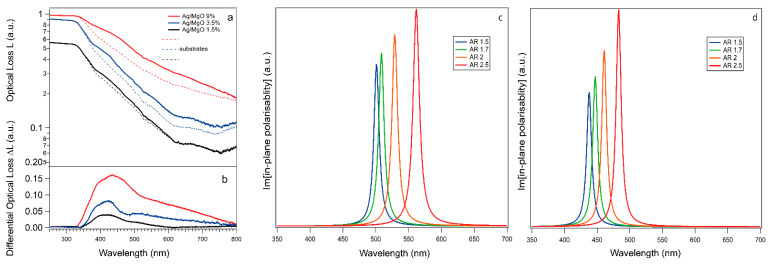
(**a**) Optical loss spectra of Ag/MgO NPs at different coverages levels. (**b**) Differential optical loss spectra of samples with different coverages of Ag/MgO NPs. The differences in signal intensity of L_sub_ (and therefore the corresponding L) for different samples may arise from small variations in contaminants (e.g., carbon), humidity and roughness. (**c**) The imaginary part of the calculated in-plane polarizability for Ag NPs having different aspect ratios embedded in a MgO matrix with refractive index n = 1.7. (**d**) The imaginary part of the calculated in-plane polarizability for Ag NPs having different aspect ratios embedded in a MgO matrix with refractive index n = 1.4. Only the in-plane component of the polarizability has been computed due to the s-polarization of the light beam.

The system optical response for different NP coverages is investigated by measuring its UV–Vis reflectivity R(λ) and transmissivity T(λ), with s-polarized light and beam incidence of 45° with respect to the surface normal. Measurements are performed on samples subdivided into four areas, on top of which four single and electrically independent cells are successively grown in the same conditions. Typically (Figure 1c (left)), three cells were functionalized with the same NP amount, while the fourth was kept pristine by proper masking, and serves as a reference. In the upper panel of Figure 6a we show the UV–Vis optical loss L, defined as L = 1 − (T + R) as a function of the wavelength, for samples corresponding to NP coverage of 1.5%, 3.5% and 9%, respectively. For each coverage, the spectrum L_sub_ of the corresponding glass/FTO/c-TiO_2_/m-TiO_2_ pristine substrate is shown for comparison.

The lower panel (Figure 6b) reports the differential optical loss, i.e., ∆L= L − L_sub_.

For 1.5% coverage, ∆L is characterized by a broad feature peaking at λ~420 nm, together with a tail extending to a higher wavelength, up to λ~700 nm. For higher coverages, the overall ∆L intensity progressively increases, the main peak slightly red shifting and becoming broader while the high-wavelength tail rises [84]. The origin of these features and their dependence on coverage can be rationalized considering that the optical loss is given by two contributions, i.e., the layer absorbance (A) and the scatter (S), the latter accounting for light diffusion induced by surface roughness [85]. The main peak observed at λ = 430 nm can be assigned to the Ag NP localized surface plasma resonance (LSPR) absorbance while the broad tail can be attributed to an enhancement in light diffusion (decreasing at longer wavelengths [86]), caused by NP-induced increasing surface roughening.

It is well known that the NP optical absorption response is highly influenced by nanoparticle shape—especially their aspect ratio (AR) [87]—as well as by the dielectric properties of the embedding material, while it is fairly independent from NP size, at least for diameters in the 5–40 nm range [88]. For bare, spherical Ag NPs, the LSPR is localized at 390 nm [47], while it shifts to higher wavelengths if NPs are embedded in a solid-state matrix [84,88], the shift being higher the larger the matrix refractive index. Following the Maxwell Garnett approach [89] (see Supplementary Materials for further details), we have computed the imaginary part of the Ag NP in-plane polarizability for different values of the NP AR [90] and of the matrix dielectric function. Indeed, as occurs in the case of other metal oxide materials, the MgO UV–Vis dielectric function is known to vary with the film thickness t, becoming smaller than in the bulk for films thinner than t~250 nm [91,92]. In Figure 6c, the imaginary part of the NP polarizability is calculated for different values of AR ranging between 1.5 and 2.5, using the dielectric constants of bulk Ag and MgO, taken from reference [93]. In this case, a red shift of the LSPR maximum from ~500 nm to ~570 nm is observed upon AR increasing. Figure 6d shows, instead, the imaginary part of the NP polarizability calculated using the smaller dielectric constant of a thin MgO layer, as taken from the literature [94]. In this case, all the spectra are blue shifted, the AR = 2 moving from 520 to 460 nm, in fair agreement with the maximum position measured for the 1.5% coverage. As previously discussed, the distribution of NP AR values is expected to broaden with increasing NP film coverages—with larger NPs having higher AR—thus explaining the observed broadening of the LSPR peak. On the other hand, particle interconnections can also influence the values of plasmonic resonance mode at high coverages, contributing to the overall broadening [95,96].

Eventually, the functionalized substrates were used to fabricate and test the overall electric performance of Ag/MgO-engineered PSCs, for different NP coverages. As shown in Figure 1c (right), the Ag/MgO NPs are localized at the interface between the ETL m-TiO_2_ layer and the mixed-cation perovskite photoactive layer.

The summary of the main performance parameters—i.e., short circuit current density (J_sc_), open circuit voltage (V_oc_), fill factor (FF) and power conversion efficiency (PCE)—is shown in Table 1 for different NP coverages; at least nine devices were tested for each coverage. The reference cell (0.0%) presents an average PCE of 16.0% ± 0.4%. The best results are obtained for an NP surface coverage of about 1.5%, which performs at an average efficiency of 16.5% ± 0.8%, corresponding to a relative improvement of ~3% in PCE. For NP coverages above 3.5%, the performance of the NP-engineered cells progressively deteriorates, possibly due to reduced transmissivity at the TiO_2_/NP interface, which reduces the fraction of light reaching the perovskite active layer [97]. For all measured devices, no perovskite degradation was visually observed, indicative of the active role played by MgO as an effective protective material of the Ag core from direct exposure to perovskite.

As shown in Table 1, the PCE enhancement observed for 1.5% coverage is related to an increase in both J_SC_ and V_OC_. The best cell electrical parameters are reported in Table 2, showing a PCE value of 17.8%, as compared to the 17.0% of the best-efficiency reference device, corresponding to an overall relative increment of about 5%. Analogous relative increments in PCE enhancement have been reported in the literature for different types of core–shell NPs deposited or embedded in the ETL. For instance, in the cases of Au@Pt@Au [44], Ag@TiO_2_@Pa [31] and Au@SiO_2_ [42], the observed increase in PCE ranges between 8 and 17%.

**Figure 7:**
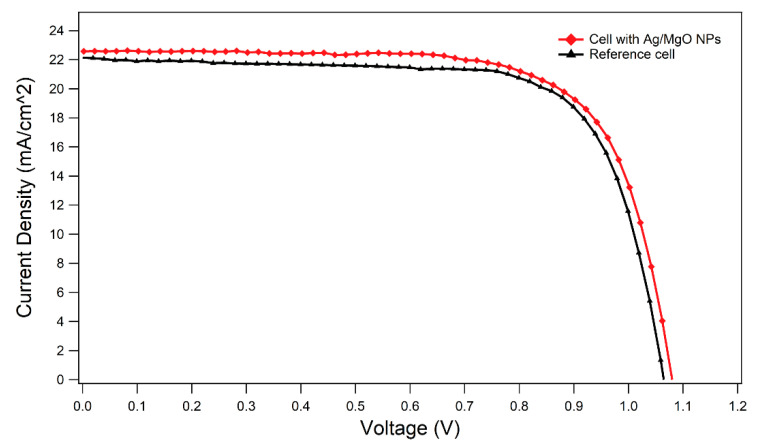
J-V curves for both the best reference cell without Ag/MgO NPs (black curve) and the best optimized device with NPs (red curve).

In Figure 7, the current density–voltage (J-V) curve for the best optimized device at about 1.5% coverage of Ag/MgO NPs is compared to the corresponding (best-performing) reference cell, at stabilized conditions for both devices.

As shown in Figure 7 and Table 2, embedding the Ag/MgO NPs atop an m-TiO_2_ layer reflected a significant enhancement of V_OC_. By following the study of Yuan et al. [34], we suggest that the improvement of V_OC_ may be due to a reduction in TiO_2_/Ag/MgO NP work function with respect to TiO_2_. In fact, under light illumination, due to the injection of carriers from Ag/MgO NPs to TiO_2_, the Fermi level is expected to increase, leading to reduced TiO_2_ work function. Finally, this reduction may enhance the built-in potential and increase the V_OC_ [98].

**Figure 8:**
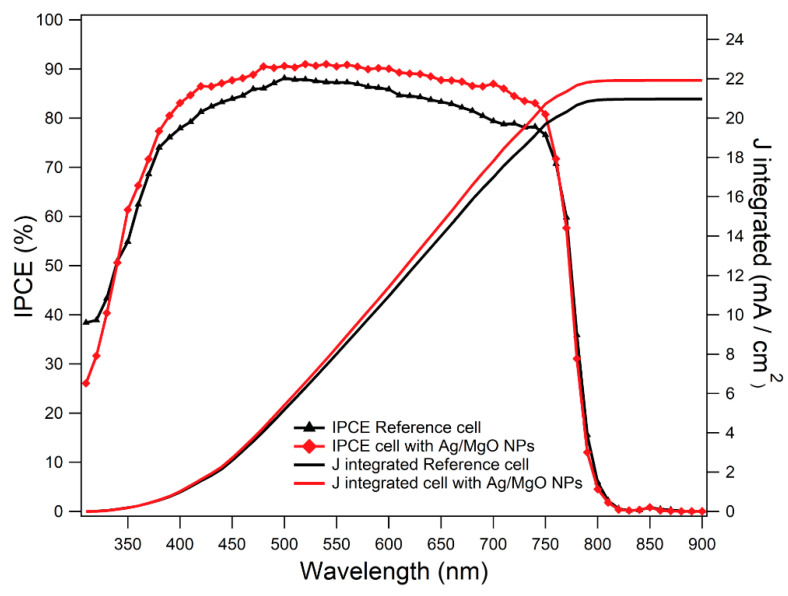
IPCE spectra for both the best-performing device with Ag/MgO NPs (red) and the best reference device (black) and their integrated current density; the J values registered are coherent with those shown in Table 2 under a solar simulator, with a percentage difference of 3% and 4.7% for the devices with and without NPs, respectively.

Moreover, in Figure 8, the IPCE spectra of the best-performing cell and of the (0%) reference cell are compared, along with the corresponding integrated current density (J_integrated_). The observed enhancement in IPCE takes place in the whole (340–760) nm optical window, in agreement with previous results reported in the literature [99,100,101]. This broadband effect has been attributed to different NP-related effects, suggesting that, besides near-field LSPR-enhanced absorption, other mechanisms such as increased far-field scattering, facilitated charge-transfer and separation [37,102] and reduction in the exciton binding energy [42,48] may play a relevant role.

## 4. Conclusions

In this work, the magnetron-based physical deposition method was applied to incorporate novel NPs in thin-film perovskite solar devices, thus probing their properties and their effect on device performance enhancement. In particular, the structural properties of Ag/MgO NPs—deposited on a suitable mesh by a co-deposition method—are investigated by HR-TEM, showing the multi-twinning icosahedral structure of the Ag core, and providing evidence (through EDXS mapping) of the actual formation of the MgO overlayer. The morphological properties of Ag/MgO NPs deposited on top of the m-TiO_2_ ETL layer by means of sequential-layer deposition were investigated through XPS, SEM and AFM, providing information on their lateral dimension and height distributions. For an NP coverage of 1.5%, the mean value of the NP lateral dimension is d = 8.0 nm while the mean height is h = 4 nm, showing that NPs are characterized by a flattened spheroidal shape, with AR~2. Furthermore, XPS measurements show the NPs’ stability against thermal treatment up to 150 °C, an important aspect to be considered for their actual use in real PSC devices. Optical loss data, taken with s-polarized UV–visible light in the 300–800 nm, are dominated by the Ag LSPR absorption band, peaking at λ~420 nm, together with a long-wavelength tail mainly attributed to diffuse scattering. Upon an NP coverage increase, the optical loss intensity increases and the LSPR peak broadens and red shifts; these changes are attributed to a change in the NP size and AR distributions and suggest the possibility of tuning the system optical response, as well as extending the plasmonic response to a broader spectral range, by changing the deposited NP amount and morphology. By embedding silver magnesia NPs at the interface between the photoactive layer (i.e., a triple-cation perovskite layer) and the m-TiO_2_ electron transport layer in thin films of PSC devices, average efficiency increases of ~3% were obtained, from 16.0% in the pristine device to 16.5% in the engineered Ag/MgO NP device at an optimized coverage value around 1.5%. It is noted that the moderate increase in device performances—as compared with other NP-engineered cells reported in the literature—becomes quite remarkable if we consider that in the tested devices, NPs were mainly localized at the surface of the mesoporous substrate. This increase is mainly related to an enhancement in the device short circuit current and open circuit voltage, while the IPCE spectrum shows that this increase is distributed on all wavelengths. This suggests, in agreement with the literature, that Ag LSPR may not be the main source of the observed efficiency enhancement, while other mechanisms such as diffuse light scattering, enhanced charge separation and reduction in exciton binding energy also have to be considered. In parallel, the improved V_OC_ suggests an upward shift of m-TiO_2_ work function due to an efficient charge transfer from Ag/MgO NPs and m-TiO_2_. This work presents our physical deposition method, based on a gas aggregation nanocluster source, as an innovative, versatile, and solvent-free strategy for plasmonic NP engineering, which will allow the exploration of different combinations of metal cores and shell/protective layer materials, aiming at the fabrication of solar devices with increased current density and efficiency.

## Figures and Tables

**Table 1:** Main parameters, with their standard deviations, for devices ranging between 0.0% and 10.0% of surface coverage in Ag/MgO NPs atop an m-TiO_2_ layer.

Ag/MgO Coverage %	J_sc_ (mA/cm^2^)	V_oc_ (V)	FF (%)	PCE (%)
0.0	21.6 ± 0.3	1.04 ± 0.03	71.1 ± 1.0	16.0 ± 0.4
1.5	22.1 ± 0.4	1.07 ± 0.03	71.5 ± 2.4	16.5 ± 0.8
3.2	21.6 ± 0.7	1.06 ± 0.02	69.7 ± 2.8	15.0 ± 1.2
8.7	12.0 ± 1.4	1.00 ± 0.03	60.2 ± 2.2	8.4 ± 1.0
10.0	7.3 ± 0.7	0.98 ± 0.01	50.9 ± 3.3	5.0 ± 0.9

**Table 2:** Main performance parameters for the best reference cell and the best optimized device with Ag/MgO NPs.

	J_sc_ (mA/cm^2^)	V_oc_ (V)	FF (%)	PCE (%)
Reference	22.0	1.05	72.2	17.0
Ag/MgO 1.5%	22.6	1.08	71.9	17.8

## Data Availability

Data is contained within the article and Supplementary Materials.

## Supplementary Materials

The following are available online at https://www.mdpi.com/article/10.3390/ma14195507/s1, Figure S1: XPS spectra of MgO on TiO_2_; Figure S2: AFM roughness analysis of the flat TiO_2_; polarizability calculation within the Maxwell Garnett model (Equation).
