# Supplementary material for: Ag/MgO Nanoparticles via Gas Aggregation Nanocluster Source for Perovskite Solar Cell Engineering
Source: Materials (Basel). 2021 Sep 23;14(19):5507. doi: 10.3390/ma14195507 (PMC8509757; doi:10.3390/ma14195507)
Supplement: Supplementary file 1 [file materials-14-05507-s001.zip › materials-1380919-supplementary.pdf]

Supplementary Material

Ag/MgO Nanoparticles via Gas Aggregation Nanocluster Source for Perovskite Solar Cells Engineering

Matteo Caleffi[1], Paolo Mariani[2], Giovanni Bertoni[3,4], Guido Paolicelli[3], Luca Pasquali[5,6,7], Antonio Agresti*[2], Sara Pescetelli[2], Aldo Di Carlo[2,8], Valentina De Renzi[1,3], Sergio D'Addato[1,3,9]*

[1] Dipartimento di Scienze Fisiche, Matematiche e Informatiche, Università di Modena e Reggio Emilia, Via Campi 213/A, 41125, Italy

[2] CHOSE, Centre for Hybrid and Organic Solar Energy, Department of Electronics Engineering, University of Rome Tor Vergata, 00133 Rome, Italy

[3] CNR – Istituto Nanoscienze, Via Campi 213/A, 41125 Modena, Italy

[4] IMEM – CNR, Parco Area delle Scienze 37/A, 43124 Parma, Italy

[5] Dipartimento di Ingegneria E. Ferrari, Università di Modena e Reggio Emilia, Via Vivarelli 10, 41125, Modena, Italy

[6] IOM – CNR, s.s. 14, Km. 163.5 in AREA Science Park, Basovizza, Trieste 34149, Italy

[7] Department of Physics, University of Johannesburg, PO Box 524, Auckland Park 2006, South Africa

[8] ISM-CNR, Istituto di Struttura della Materia, Consiglio Nazionale delle Ricerche, 00133 Rome, Italy

[9] EN&TECH, Università di Modena e Reggio Emilia, Italy

***** Correspondence: matteo.caleffi@unimore.it; antonio.agresti@uniroma2.it.

XPS of MgO on TiO_2

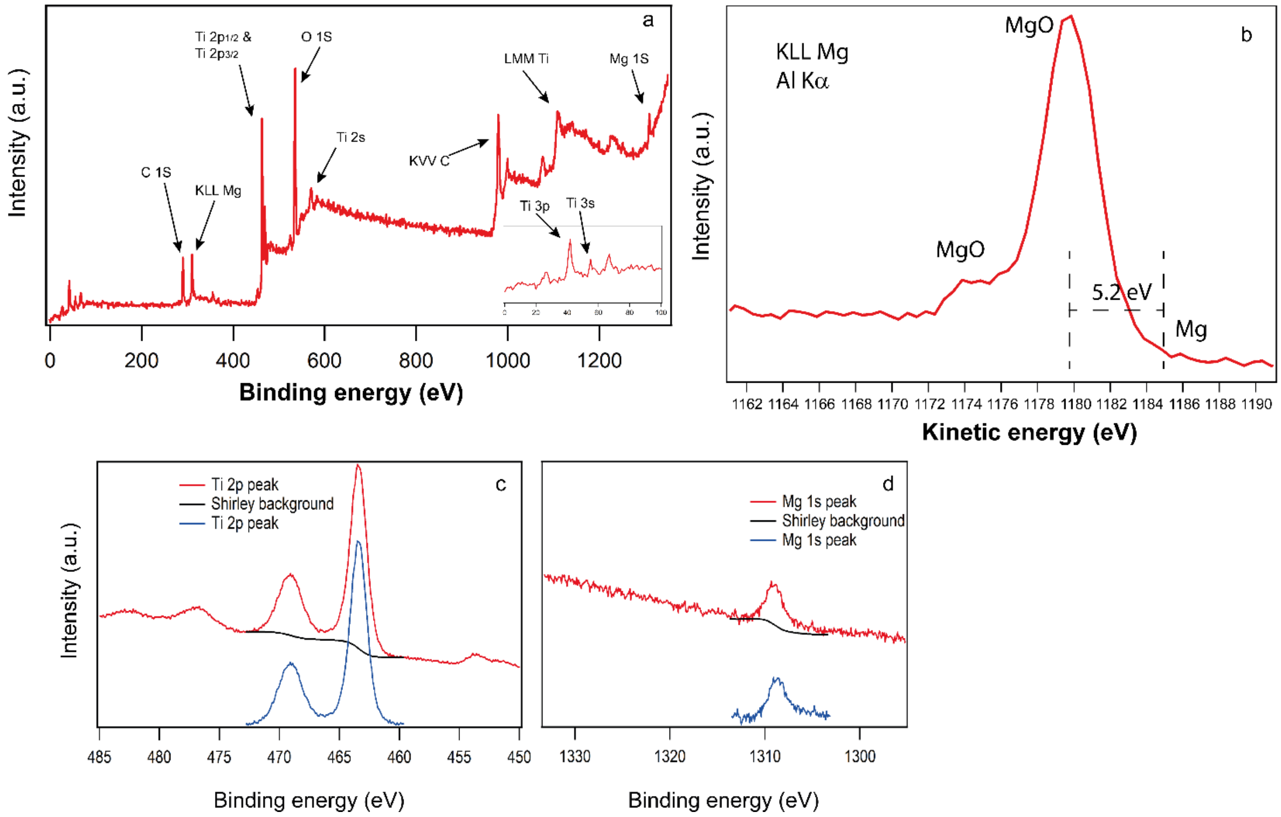

Figure S1: XPS (a) wide spectrum of the TiO_2 sample with MgO deposited on the surface, **(**b) Mg KLL, (c) Ti 2p core levels and (d) Mg 1s core levels with background subtractions.

AFM

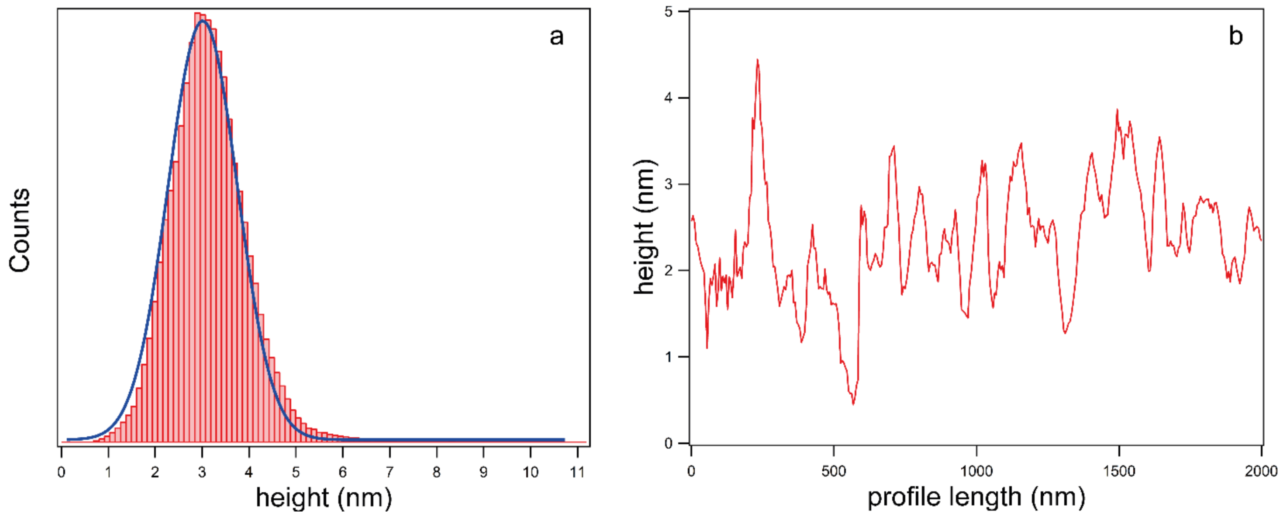

Figure S2: AFM (a) Height distribution of the substrate roughness (flat TiO_2) and (b) height of a profile line of 2 μm length.

Calculation of polarizability

The in-plane polarizability of a system made of isolated Ag oblate spheroids (a = b > c) embedded in a magnesia (MgO) matrix is calculated using Maxwell Garnett theory:

$$\alpha_{//}(\omega) \propto \frac{\varepsilon_{Ag}(\omega) - \varepsilon_{MgO}}{\varepsilon_{MgO} + L_{//}(\varepsilon_{Ag}(\omega) - \varepsilon_{MgO})}$$

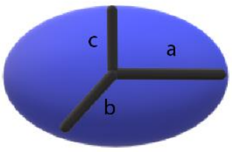

where $\varepsilon_{Ag}(\omega)$ is the dielectric function of Silver, ε_{MgO} is the dielectric function of Magnesium Oxide, $L_{//}$ is the depolarisation factor of the in-plane direction. Different values of ε_{MgO} have been used according to what found in literature for thin layers of MgO.
